# Long Noncoding RNA GAS5 Contained in Exosomes Derived from Human Adipose Stem Cells Promotes Repair and Modulates Inflammation in a Chronic Dermal Wound Healing Model

**DOI:** 10.3390/biology11030426

**Published:** 2022-03-11

**Authors:** Rekha S. Patel, Sabrina Impreso, Ashley Lui, Gitanjali Vidyarthi, Paul Albear, Niketa A. Patel

**Affiliations:** 1 James A. Haley Veteran’s Hospital, 13000 Bruce B Downs Blvd, Tampa, FL 33612, USA; Sabrina.Impreso@va.gov (R.S.P.); impresab@gmail.com (S.I.); gitanjali.vidyarthi@va.gov (G.V.); paul.albear@va.gov (P.A.); 2 Department of Molecular Medicine, University of South Florida, Tampa, FL 33612, USA; ashleylui@usf.edu

**Keywords:** long noncoding RNA (lncRNA), GAS5, exosomes, inflammation, wound healing, human adipose stem cells, chronic wounds, lipopolysaccharide (LPS), human dermal fibroblast (HDF)

## Abstract

**Simple Summary:**

Wounds due to cuts, lacerations, or surgical incisions undergo healing through a highly regulated process. Occasionally, the skin is unable to heal in a timely manner, leading to chronic wounds and related sequelae, such as scarring, risk of infections on open wounds, and—as a growing body of evidence attests—psychological impact on the individual. In addition, certain diseases, such as diabetes, obesity, and cancer, are characterized by an ongoing state of very low-grade inflammation. This underlying inflammation substantially hinders wound healing. To improve the outcome of chronic wounds, we harvested the potential of exosomes (nanovesicles) secreted from human adipose stem cells. We demonstrate that exosomes are efficiently taken up by skin cells and promote healing by significantly accelerating wound closure time. To understand the mechanism by which exosomes promote wound healing, we identified an RNA called GAS5 that is a driver of the regenerative properties of exosomes. Additionally, we identified the inflammation pathways that are regulated by GAS5 to promote the healing of wounds. Such a determination is essential to move exosome therapy into the clinic. In conclusion, our results demonstrate that exosomes harvested from human adipose stem cells accelerate the healing of chronic recalcitrant wounds and thus have a tremendous therapeutic potential in wound healing.

**Abstract:**

Chronic recalcitrant wounds result from delayed or slowed healing processes. Underlying inflammation is a substantial risk factor for impaired dermal wound healing and often leads to chronic wound-related sequelae. Human adipose stem cells (hASCs) have shown tremendous potential in regenerative medicine. The goal of this project was to improve the outcome of chronic wounds by harvesting the exosomes from hASCs for therapeutic intervention. The results demonstrate that long noncoding RNA GAS5 is highly enriched in hASC exosomes and, further, that GAS5 is central to promoting wound repair in vitro. To evaluate the outcome of wound healing in a chronic low-grade inflammatory environment, lipopolysaccharide-treated HDF cells were evaluated for their response to hASC exosome treatment. Ingenuity pathway analysis identified inflammation pathways and genes affected by exosomes in a GAS5-dependent manner. Using siRNA to deplete GAS5 in HDF, the results demonstrated that Toll-like receptor 7 (TLR7) expression levels were regulated by GAS5. Importantly, the results demonstrate that GAS5 regulates inflammatory pathway genes in a chronic inflammation environment. The results presented here demonstrate that hASC exosomes are a viable therapeutic that accelerate the healing of chronic recalcitrant wounds.

## 1. Introduction

Dermal wounds, which are breaks in the structure of skin due to cuts, lacerations, or incisional wounds post-surgery, are healed through a regulated repair process. At times, healing of wounds is delayed or slowed, resulting in chronic recalcitrant wounds. Underlying dermal inflammation is a substantial risk factor for impaired wound healing and often leads to chronic wound-related sequelae. Human adipose stem cells (hASCs) have shown tremendous potential in regenerative medicine. The stem cell antigens and markers of hASCs are similar to the mesenchymal stem cells isolated from the umbical cord and bone marrow. hASCs have significant advantages over other sources of stem cells, primarily due to their abundance and easily accessible locations. The regenerative potential of hASCs is dependent on secreted bioactive material which is packaged and released in extracellular vesicles. We sought to improve the outcome of injury and promote wound healing by harvesting the secretome of hASCs for therapeutic intervention. We have mastered the isolation of hASCs from adipose tissue, where hASCs reside in the stromal vascular fraction. We have characterized the stem cell antigens and markers and evaluated their renewal capacity and differentiation potential and we have shown that the hASC secretome contains exosomes which are small extracellular vesicles (30–150 nm) that regulate fundamental cellular functions in recipient cells. While all cells secrete exosomes, their contents differ significantly. Our lab has characterized hASCs, their secretome collected as conditioned media (CM), and their exosomes [1].

The advantages of the hASC exosome are that it provides a cell-free regenerative approach over other therapies, such as nanoparticles or stem cells. The natural biocomponents of exosomes are not toxic. We previously demonstrated that an RNA component of exosomes drives its regenerative therapeutic ability. We demonstrated the presence of several long noncoding RNAs (lncRNA) in the secretome of hASCs, and additionally our studies indicated specific lncRNAs that were packaged in large amounts in exosomes [1,2,3]. The lncRNAs are packaged into exosomes to prevent their degradation by nucleases. Exosomes release the lncRNA cargo into the target cells where it regulates gene expression and influences the genomic landscape. 

Here, we evaluated the outcome of wound healing with treatment with hASC exosomes using human dermal fibroblasts (HDF). Our results showed a significantly accelerated wound closure time, supporting the use of hASC exosomes in dermal wound healing. Previously, using RNAseq to analyze the lncRNA content of hASC exosomes, the results identified lncRNA growth-arrest specific-5 (GAS5) and metastasis-associated lung adenocarcinoma transcript 1 (MALAT1), which were highly enriched in exosomes [1,4]. In our prior studies, we initially started evaluating MALAT1 as it is a strong regulator of expression of several genes. Our extensive previous studies have shown that MALAT1 accounted for at least 50% of wound healing by exosomes and we identified the pathways and alternative splicing events affected by MALAT1 contained in exosomes [2,5]. However, the role of GAS5 contained in hASC exosomes in wound repair is not yet deciphered. Hence, we sought to evaluate whether exosomal GAS5 was essential for wound repair. Further, wound infection is a significant clinical problem and thus we evaluated wound healing under lipopolysaccharide (LPS)-induced chronic inflammation and the efficacy of treatment with hASC exosomes. Using ingenuity pathway analysis (IPA), we identified the inflammation signaling pathway mediators and Toll-like receptors that were implicated in wound healing by hASC exosomes under conditions of chronic low-grade inflammation. Further, we identified the genes whose expression changed in a GAS5-dependent manner. These results elucidate the mechanisms by which hASC exosomes promote healing in chronic wounds.

## 2. Materials and Methods

### 2.1. Cell Culture and Treatments

Primary human dermal fibroblasts (HDF) were purchased from Sciencell (catalog# PCS-201-012) and passaged as preconfluent cultures in fibroblast media (Sciencell catalog# 2301). Human adipose stem cells (hASC) were purchased from Zenbio (catalog #ASC-F) and grown to confluency in its media (Zenbio, catalog #PM-1; also called stem cell media as it maintains the stemness of hASC). The hASCs were characterized by detecting stem cell antigens and markers (positive for CD44, CD90, CD115 and negative for CD31, CD45, CD14) using flow cytometry as described by us in our prior publication [1]. The hASC were cultured up to 2 passages to obtain exosomes from its secretome (described in 2.2) to evaluate exosomes as a therapeutic for treatment of dermal wounds. All cells were grown at 37 °C and 10% CO_2_. To induce inflammation, LPS was added to cells [2,3,4]. For acute treatment experiments, 5 ng/mL LPS (Sigma, Kawasaki, Japan) was added to 90% confluent HDF for 6 h and then medium was changed to remove LPS. For chronic treatment experiments, 5 ng/mL LPS was added to 90% confluent HDF for 6 h to induce inflammation. LPS was maintained in medium, and cells were treated with or without hASC exosomes for 4 days, as indicated in the experiments. Oxidative stress is a key player in the pathogenesis of non-healing wounds. Then, 100 μM of H_2_O_2_ (Fisher catalog# H323-500) was added for 1 h to HDF cells to induce oxidative stress, after which the medium was changed to remove H_2_O_2_ and the cells were treated with or without hASC exosomes.

### 2.2. Exosome Isolation from Human Adipose Stem Cells (hASCs)

The hASC media (Zenbio, catalog #PM-1) was centrifuged at 100,000× *g* for 60 min to remove extracellular vesicles and exosomes from media. The hASCs at 90% confluency were then cultured in exosome-free hASC media to ensure that all exosomes isolated were derived from hASCs. After 48 h, the conditioned medium (CM) from 8 × 10^6^ hASC was collected. Exosomes were isolated from CM as previously described by our lab [1,5]. Briefly, conditioned media derived from hASCs was collected after 48 h and centrifuged at 3000× *g* for 15 min to remove dead cells. ExoSpin™ (Cell Guidance system; Catalog EX05) reagent was added to the CM and incubated for 20 min at room temperature. Following centrifugation at 1500× *g* for 30 min to remove cellular debris, the supernatant was applied to the top of ExoSpin columns and centrifuged at 50× *g* for 60 s. Exosomes were eluted in PBS by centrifugation at 50× *g* for 60 s. Nanoparticle tracking analysis with NanoSight (NTA3.1, Build 3.1.46 RRID SCR-014239) was used to analyze peak diameter and the concentration of exosomes obtained from 106 hASC. Analysis showed exosome size to be 94 ± 7 nm.

### 2.3. Transfection of hASCs

To label hASC exosomes, 1 × 10^6^ hASCs (Zenbio, catalog #ASC-F) were trypsinized and cell pellets were transfected with either 2 μg mCherry (Addgene, 128744) or 2 μg GFP-pmax (included in Nucleofector kit) using a Nucleofector^®^ kit (Lonza, catalog #VPE-1001). The cell/DNA solution was transferred to a cuvette and the program initiated (0.34 kV, 960 μF). Medium (500 μL) was added immediately and cells were gently transferred to 100 mm plates and allowed to grow for 48 h. Exosomes were verified to contain mCherry by PCR using sense primer 5′-CAGGACGGCGAGTTCATCTA-3′ and antisense 5′-GTCTTGACCTCAGCGTCGTA-3′; and verified to contain GFP by PCR using sense primer 5′-AGGCGTGTACGGTGGGAG-3′ and antisense 5′-CTACAAATGTGGTATGGCTGA-3′. To deplete MALAT1 from hASC exosomes, 1 µM MALAT1 antisense oligonucleotide (ASO; ID: 39524 ASO from Ionis Pharmaceuticals, validated for specificity and designed for efficient uptake by cells as demonstrated by us previously [5]), was added to the hASCs and incubated for 48 h. To deplete GAS5 from hASC exosomes and from HDF, 25 nM GAS5 siRNA (ThermoFisher/Ambion catalog# n520782) was transfected into cells using RNAiMax (ThermoFisher catalog #13778075) for 48 h. The siRNA, selected from four siRNAs that target separate areas on GAS5 and evaluated for optimal knockdown of expression of GAS5, was validated for efficacy, non-toxicity, and specificity to eliminate off-target effects, as described by us previously [5,6,7]. A negative control siRNA (Thermo Fisher catalog #4404021), RNAiMax alone (transfection control) and an untreated control were included in all experiments. The expression levels and knockdown of MALAT1 and GAS5 in the exosomes were verified using human MALAT1 and GAS5 primers in qPCR and absolute levels were determined as described below in Section 2.4.

### 2.4. Real-Time Quantitiative Polymerase Chain Reaction (RT-qPCR)

Total RNA was isolated from 1 × 10^6^ HDF using TrizolTM (Thermo Fisher Scientific, Waltham, MA, USA) as per the manufacturer’s instructions. A quantity of 1 µg of RNA (260/230 > 1.8 and 260/290 > 1.8) was used to synthesize cDNA using ReadyScript^TM^ synthesis mix (Sigma RDRT). Real-time qPCR was then performed in triplicate using 1 μL of cDNA and Maxima SYBR Green/Rox qPCR master mix (Thermo Scientific). Amplification was performed on the ViiA 7 (ABI). Primers were purchased from Origene (qSTAR qPCR primer pairs). These primer pairs were pre-designed, validated, and tested for specificity to eliminate off-target effects. Primers had a Tm of 60–61 and an amplicon of 95–140 bp. The optimal primer concentration was determined from a range of 50–900 mM. The final concentration of each primer pair was selected to ensure efficiency and specificity for its target in HDF based on the dissociation curve that showed a single, sharp peak indicating that the primers amplify one specific target (described by us previously in [8].) Primers used in qPCR included GAS5 S 5′-CTTCTGGGCTCAAGTGATCCT-3′, GAS5 AS 5′-TTGTGCCATGAGACTCCATCAG-3′, MALAT1 S 5′-CTTCCCTAGGGGATTTCAGG-3′, MALAT1 AS 5′-GCCCACAGGAACAAGTCCTA-3′, IL1β S 5′-CCACAGACCTTCCAGGAGAATG-3′, IL1β AS 5′-GTGCAGTTCAGTGATCGTACAGG-3′, IL6S 5′-AGACAGCCACTCACCTCTTCAG-3′, IL6 AS 5′-TTCTGCCAGTGCCTCTTTGCTG-3′, TLR4 S 5′-CCCTGAGGCATTTAGGCAGCTA-3′, TLR4 AS 5′-AGGTAGAGAGGTGGCTTAGGCT-3′, TLR7 S 5′-CTTTGGACCTCAGCCACAACCA-3′, TLR7 AS 5′-CGCAACTGGAAGGCATCTTGTAG-3′, IR S 5′-GTTTTCGTCCCCAGGCCATC-3′, IR AS 5′-CCAACATCGCCAAGGGACCT-3′, ITGB2 S 5′-AGTCACCTACGACTCCTTCTGC-3′, ITGB2 AS 5′-CAAACGACTGCTCCTGGATGCA-3′, IL18 S 5′-GATAGCCAGCCTAGAGGTATGG-3′, IL18 AS 5′-CCTTGATGTTATCAGGAGGATTCA-3′, CCL17 S 5′-TTCTCTGCAGCACATCCACGCA-3′, CCL17 AS 5′-CTGGAGCAGTCCTCAGATGTCT-3′, and GAPDH S 5′ GATCATCAGCAATGCCTCCT-3′ and GAPDH AS 5′-TGTGGTCATGAGTCCTTCCA-3′. For absolute quantification using SYBR Green qPCR, a standard curve was generated for each gene in every assay. For this, 100–0.4 ng of RNA from HDF was reverse-transcribed as described above. The resulting cDNA was used to obtain a standard curve correlating the amounts with the threshold cycle number (Ct values). A linear relationship (*r*_2_ > 0.96) was obtained for each gene. Plate set up included the standard series, no template control, and no reserve transcriptase control. The dissociation curve was analyzed for each sample. Absolute quantification (AQ) for expression levels of individual transcripts was calculated by normalizing the values to GAPDH. Relative quantity (RQ) was determined by the ΔΔCt method with GAPDH as the endogenous control and control sample as the reference calibrator. In addition, to validate the qPCR data, 1 µL of cDNA was amplified with the primer pairs, including GAPDH endogenous control primers, using JumpStart ReadyMix (Sigma P0982); products were run on a 1% agarose gel and stained with ethidium bromide for visualization of bands and imaged in ProteinSimple FluorChem M. GAPDH stability under different treatments was verified by visualizing a single band per sample followed by densitometric analysis of the bands across treatments using the integrated AlphaView^®^ software 3.5.0 (ProteinSimple, San Jose, CA, USA). 

### 2.5. Western Blot Analysis

**Figure 1:**
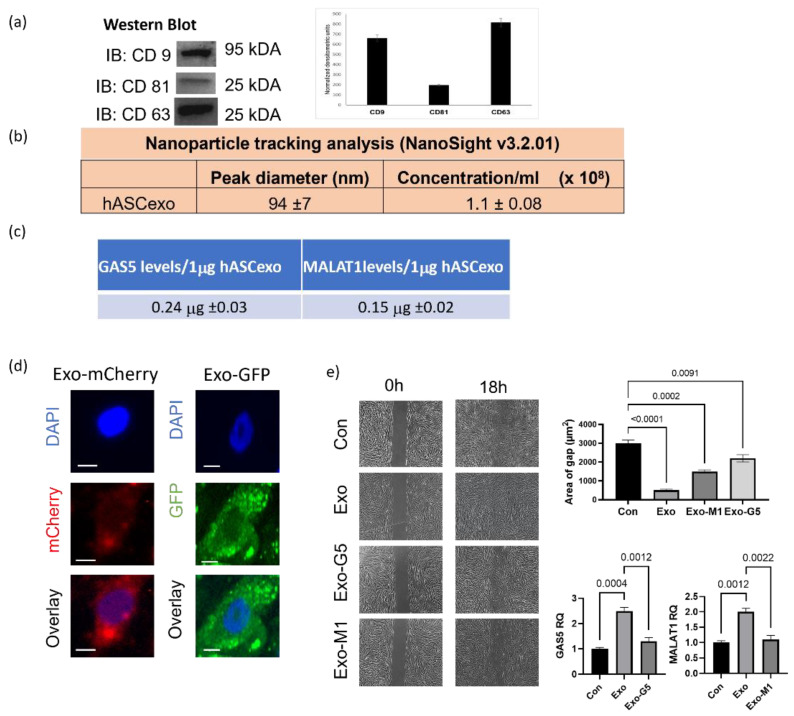
Exosomes isolated from hASCs were verified by (**a**) western blot for hASC exosomal tetraspanin markers using antibodies against CD9, CD63, and CD81. The bands are representative of results obtained from experiments repeated five times. The graph represents ±SEM densitometric units. (**b**) The size and purity od hASC exosomes were evaluated using NanoSight v3.2.01 and (**c**) levels of long noncoding RNAs GAS5 and MALAT1 by absolute quantification by qPCR per 1 µg of exosomes across batches. (**d**) 1µg of mCherry or GFP overexpression plasmids were transfected into hASCs and exosomes were isolated from conditioned media. HDF cells were treated with 1 µg of exosomes and imaged using the Keyence microscope after 24 h showing uptake of hASC exosomes carrying mCherry or GFP (scale bar 20 µm, *n* = 3). (**e**) HDF cells were grown in a 35mm plate with Ibidi μ-inserts to generate consistent gaps. Inserts were removed and HDF cells were treated with exosomes (Exo) or exosomes depleted of GAS5 (Exo-G5) or depleted of MALAT1 (Exo-M1). Gap was imaged at time 0 and re-imaged after 18 hours. Wound gap was measured using Image J and area was calculated in µm^2^ (*n* = 3). Statistical analysis was performed by one-way ANOVA and significant *p*-values (<0.05) are indicated on graph.

Protein lysates were obtained from the hASC exosome preparations using lysis buffer containing protease inhibitors (Cell signaling #9803s). The bicinchoninic acid (BCA) protein method was used to quantitate total protein in the samples. A quantity of 40 μg of lysates were separated by SDS-PAGE using 8–15% acrylamide gels, then electrophoretically transferred to nitrocellulose membranes (at 90 V for 1 h), blocked with 5% bovine serum albumin in Tris-buffered saline containing 0.1% Tween 20 (TBST) for 1 h. Membranes were washed thrice with TBST. To detect the presence of tetraspanins (markers for exosomes), the membrane was incubated overnight at 4 °C with either anti-CD9 (Abcam ab236630; 1:1000), anti-CD63 (Abcam ab134045; 1:1000), or anti-CD81 (Abcam ab79559; 1:1000). After incubation with anti-rabbit IgG-HRP (1:3000) for 30 min, enhanced chemiluminescence (Pierce) was used for detection and images were digitally captured using ProteinSimple FluorChem M. Experiments were repeated thrice, and representative bands demonstrating the presence of CD9, CD63, and CD81 on exosome preparations are shown in Figure 1a. Densitometric analysis was performed using the AlphaView^®^ software 3.5.0 (ProteinSimple, San Jose, CA, USA). 

### 2.6. Inflammation Array and Analysis

Total RNA was extracted from HDF cells and cDNA was generated as described above in Section 2.4. The cDNA was amplified using the human inflammation array (Qiagen catalog# PAHS-077Z) according to the manufacturer’s instructions. The array consists of 90 inflammation pathway genes, housekeeping genes, and positive and negative controls. The data were analyzed using Qiagen/GeneGlobe’s Enterprise Data Analysis Solutions and differentially expressed genes compared to controls were identified. Data were further analyzed using ingenuity pathway analysis (IPA) to identify direct and indirect relationships and a comparison analysis was performed to identify causal networks and signaling pathways. Results were filtered to human fibroblast data and z-scores ≥ ±2 were considered. 

### 2.7. Seahorse Metabolic Assay

HDF cells were plated into a poly-D-lysine-coated Seahorse XFp cell culture mini-plate (Agilent Technologies, Santa Clara, CA, USA) at a density of 4000 cells per well as determined by optimization cycles. To mimic oxidative stress in HDF and the response to treatment with exosomes, HDF were treated acutely with 100 μM H_2_O_2_ for 1 h followed by a change of cell culture medium and treatment with 1 μg of exosomes (Exo) or GAS5-depleted exosomes (Exo-G5) for 18 h. The media was then changed to Seahorse XF Media (supplemented to 100 mM pyruvate, 200 mM glutamine, and 2.5 M glucose) before being imaged on the Keyence microscope to calculate cell count for normalization. Mitochondrial function in live cells was determined by performing the Cell Mito Stress Test [9] using Seahorse XF (Agilent). The plate was incubated in a non-CO_2_ incubator at 37 °C for 1 h. Seahorse sensor cartridges were prepared, and solutions were loaded into ports as described for the XFp Mito Stress Test (100 μM oligomycin, 100 μM fluoro-carbonyl cyanide phenylhydrazone FCCP, and 50 μM antimycin A/rotenone were added to cells. The experiment was performed using the Seahorse XFp Analyzer. Oxygen consumption rates were measured at intervals of approximately 5–8 min. The measurements were normalized to cell counts and data were analyzed using the Agilent Wave software.

### 2.8. AOPI Cellular Survival Assay

HDF cells were grown in a 12-well plate and at 90% confluency treated with 5 ng/mL LPS for 6 h followed by exosome treatment, as described in experiments. Cells were then trypsinized and washed once with PBS. The cell pellet (containing one million cells) was resuspended in 500 μL PBS and fixed by slow, drop-wise addition of 4.5 mL ice-cold 70% ethanol while gently vortexing. Samples were incubated overnight at 4 °C to complete fixation and then stored at −20 °C until stained. A fresh solution of propidium iodide (1 mg/mL, ViaStainTM CS1-0109) and RNase A (2 mg/mL, Thermo Fisher EN0531) was diluted in water. Fixed cells were centrifuged at 1000 rpm for 5 min. The cell pellet was washed twice with PBS and the pellet was resuspended in 50 μL PI/RNase A solution and incubated at room temperature for 5 min. One milliliter PBS was added, and samples were divided to create unstained negative control for analysis. Acridine orange (ViaStainTM CS2-0106) was added (1:1) to samples for staining and incubated at 37 °C for 30 min then analyzed on the Nexcelom K2 cellometer.

### 2.9. Cell Migration by Scratch Assay

To evaluate the efficacy of hASC exosomes in dermal wound healing, we performed the scratch assay, a widely used wound healing assay, in HDF. HDF cells were grown to confluency in 35 mm dishes with μ-Dish inserts (Ibidi solutions™, 81176) to make consistent and reproducible 500 μm gaps. Cells were treated with hASC exosomes, as described in each experiment, then the inserts were removed and cell migration was imaged on the Keyence BZx-810 microscope at 4× magnification. Images were taken at the same location saved into the Keyence BZx-810 microscope with images taken at 0 and 18 h or 0, 18, and 24 h, as indicated in the individual experimental setup. The open areas between lateral cell boundaries were quantified using Image J software with the plugin Wound_healing_size_tool. 

### 2.10. Three-Dimensional Wound Assay

To mimic the microenvironment of skin, we used a 3D wound healing model. A collagen scaffold was prepared in 35 mm plates. To do so, for each well, 800 µL of collagen type I (Sigma cat#C3867) was reconstituted with 100 µL ice cold 10× DMEM (+Phenol Red) and mixed by slow and gentle pipetting on ice. Color change of DMEM was observed from red to yellow, indicating the acidity of the solution. Then, 10× reconstitution buffer was made fresh (2.2 g of sodium bicarbonate and 4.8 g of HEPES in 100 mL of distilled water, filtered through a 0.22 µm vacuum filter). A quantity of 100 µL of 10× reconstitution buffer was gently mixed into collagen/DMEM solution. Color change of DMEM was observed from yellow to light pink, and pH was verified to be within the range of 7.1–7.4. The mixture was incubated on ice for 5 min, then collagen was spun at 10,000 rpm 3 min at 4 °C to remove air bubbles. Collagen was pipetted into a 24-well plate and permitted to polymerize at 37 °C for 2 h. HDF cells were plated at 300,000 cells per well and grown to confluency. A circular wound was generated using a cut pipet tip punched into collagen. The cavity was filled with collagen to generate a 3D circular wound free of cells. The 3D wound model was kept at 37 °C. The wound gap was imaged using Keyence BZx-810 every 24 h. Wound closure was calculated each day using Keyence’s cell migration assay with Hybrid cell count software to calculate wound gap area.

### 2.11. Immunocytochemistry

HDF cells were plated into a 12-well plate, lipopolysaccharide (LPS; 5 ng/mL) was added for 6 h, and the medium was changed to remove LPS and 1 μg Exo or Exo-G5 was added for 18 h. Cells were fixed with 4% paraformaldehyde for 30 min at room temperature and blocked with 1% bovine serum albumin in PBS for 1 h. Cells were incubated overnight in primary antibody against Ki67 (1:1000, Novacastra). Cells were then washed with PBS and rocked in the dark for 1 h in Alexafluor 488 secondary antibody (1:1000, Invitrogen). Cells were rinsed with PBS thrice for 30 s each and stained with DAPI mounting media. Images were captured using a Keyence BZx-810 microscope and analyzed using Keyence Analyzer software. 

### 2.12. Statistical Analysis

All experiments were repeated 3–5 times as biological replicates and experimental samples run in triplicate to ensure the reproducibility of results. Analyses were performed using PRISMTM software and analyzed using a two-tailed Student’s *t*-test and one-way or two-way ANOVA, as indicated in figure legends. * *p* < 0.05, ** *p* < 0.01, and *** *p* < 0.001 were used as significant measures.

## 3. Results

### 3.1. Exosomes from hASCs Promote Wound Healing

Human adipose stem cells (hASCs) and their secretome show tremendous potential in healing wounds. We sought to evaluate the role of the smallest nanovesicles called exosomes secreted by hASCs. Exosomes were isolated from the conditioned media of hASCs. Western blot analysis (Figure 1a) was performed to validate the presence of the tetraspanin markers of human exosomes CD9, CD63, and CD81. To evaluate the purity, concentration, and size of exosomes, NanoSight and its analysis software NTA was used (Figure 1b). Our previous studies demonstrated that long noncoding RNAS (lncRNAs) contained in hASC exosomes are crucial to the regenerative properties of exosomes. Using RNAseq and qPCR, our results had shown that amongst the noncoding RNAs, the lncRNAs GAS5 and MALAT1 were highly enriched in hASC exosomes [1]. Hence, using qPCR we determined the levels of GAS5 and MALAT1 in every batch of hASC exosomes used in these experiments. Figure 1c shows the expression levels of GAS5 and MALAT1 per microgram of exosomes and the tolerance ranges of levels of GAS5 and MALAT1 contained in the preparations of hASC exosomes. 

To determine uptake of hASC exosomes by human dermal fibroblasts (HDF), 1 μg of exosomes labeled with either mCherry or GFP pmax was added to HDF for 24 h. The cells were then imaged using a Keyence microscope. The results (Figure 1d) demonstrates that hASC exosomes are efficiently taken up by HDF and are equally distributed within the cytoplasm and nucleus. 

Next, we sought to evaluate the efficacy of hASC exosomes in dermal wound healing and further determine whether MALAT1 or GAS5 contained in the hASC exosomes affected the ability of hASC exosomes to promote the repair of wounds. To do so, we performed the scratch assay, a widely used wound healing assay, in HDF. GAS5 or MALAT1 was depleted from hASC, as described in the section on Materials and Methods, and exosomes were isolated from the conditioned media. HDF cells were plated in 35 mm dishes with μ-inserts to create scratches (cell-free gaps) mimicking dermal wounds. After 24 h, the insert was removed and HDF were treated with 1 μg of hASC exosomes (Exo), GAS5-depleted exosomes (Exo-G5), or MALAT1-depleted exosomes (Exo-M1), as indicated in the experiments. Images were taken using a Keyence BX810 microscope at 0 h and 18 h. Quantification of wound healing was achieved by ACAS image analysis software (Ibidi solutions™). Our results (Figure 1e) demonstrate that exosomes closed the wound gap 84% faster compared to control. Compared to exosomes treatment (Exo), depleting GAS5 (Exo-G5) significantly reduced the wound healing outcome, thereby lowering the efficacy of exosomes. Exo-M1 treatment also decreased healing in concurrence with our previously published data [10]. These results demonstrate that exosome treatment accelerated wound closure and, further, that depleting either GAS5 or MALAT1 from exosomes substantially attenuated exosome-mediated wound healing. Our previous studies have demonstrated the role of MALAT1. In this project, we focused on elucidating the role of GAS5 contained in hASC exosomes in dermal wound healing.

### 3.2. hASC Exosome Treatment Alleviates Oxidative Stress in HDF

**Figure 2:**
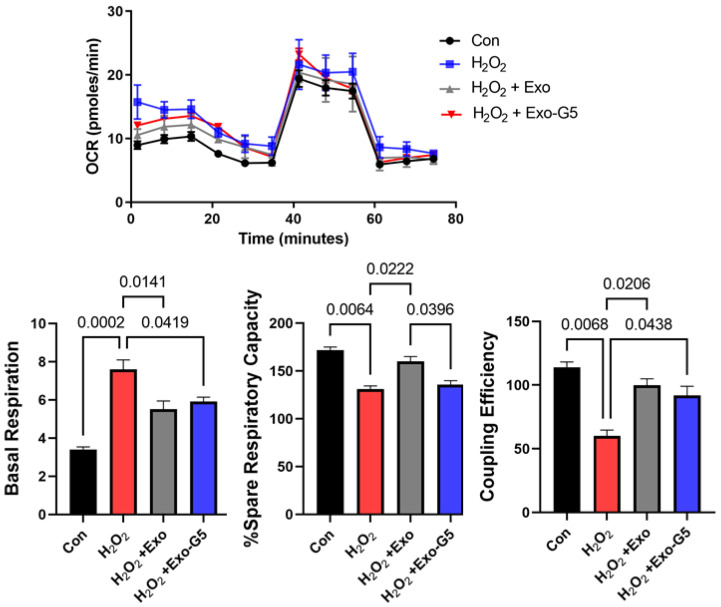
HDF cells were seeded in a Seahorse XFp miniplate and treated with 100 μM H_2_O_2_ for 1 h, followed by medium change and treatment with hASC exosomes (Exo) or exosomes depleted of GAS5 (Exo-G5) for 18 h. A Mito Stress Test Assay was performed according to the manufacturer’s instructions and repeated three times. The readings were normalized to the protein content of each well. Seahorse Wave software was used for analysis of oxygen consumption rate (OCR), basal respiration, percent spare respiratory capacity and coupling efficiency (*n* = 3). Statistical analysis was performed by one-way ANOVA and significant *p*-values (<0.05) are indicated on the graph.

Low concentrations of reactive oxygen species are integral to wound healing in the skin as they aid to fight any invading microorganisms and promote cell survival pathways. However, oxidative stress is produced by high levels of reactive oxygen species, such as H_2_O_2_. Oxidative stress is a key player in the pathogenesis of non-healing wounds. To mimic oxidative stress in HDF and the response to treatment with exosomes, HDF were treated acutely with a high concentration of H_2_O_2_. A quantity of 100 μM of H_2_O_2_ was added to HDF for 1 h, followed by change in cell culture medium and treatment with 1 μg of exosomes (Exo) or GAS5-depleted exosomes (Exo-G5) for 18 h. Mitochondrial function in live cells was determined by performing the Cell Mito Stress Test [9] using Seahorse XF (Agilent). Results (Figure 2) showed that H_2_O_2_ treatment increased basal oxygen consumption rate (OCR) due to an increase in proton leakage along with decreased respiratory capacity and decreased coupling efficiency. Treatment with Exo significantly reversed the detrimental effects of H_2_O_2_ on OCR, while treatment with Exo-G5 showed a lower ability to reverse the damage caused by H_2_O_2_ compared to Exo.

### 3.3. GAS5 Contained in hASC Exosomes Is Critical for Regeneration in Wound Models

Next, we sought to evaluate the effect of wound healing outcomes under inflammatory conditions with exosome treatment. To mimic acute inflammation, lipopolysaccharide (LPS; 5 ng/mL) was added for 6 h, medium was changed to remove LPS, and 1 μg Exo or Exo-G5 was added for 18 h. This experimental setup was utilized in the following series of evaluations of the efficacy of hASC exosomes in wound healing. 

**Figure 3:**
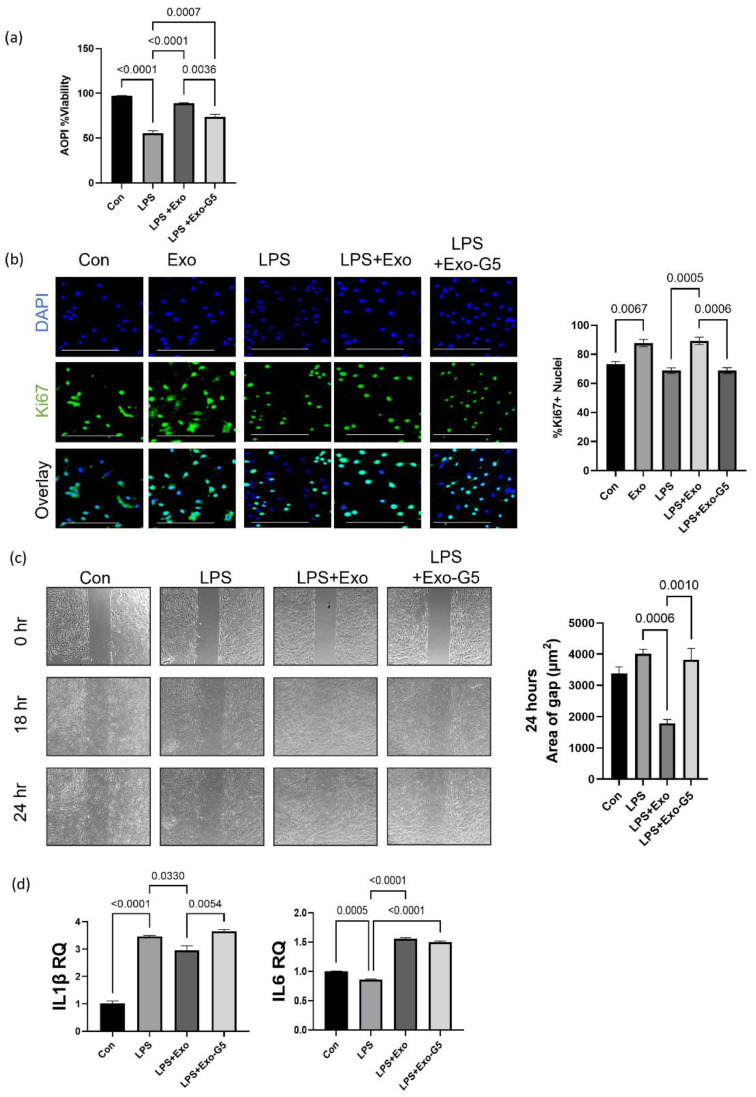
HDF cells were treated with 5 ng/mL LPS for 6 h and the medium was changed, followed by treatment with 1 μg hASC exosomes (Exo) or exosomes depleted of GAS5 (Exo-G5) for 18 h. (**a**) Acridine orange (AO) and propidium iodide (PI) dual staining was used to determine the viability of HDF cells (*n* = 3). Statistical analysis was performed by one-way ANOVA and significant *p*-values (<0.05) are indicated on the graphs. (**b**) Immunocytochemistry was performed using Ki67 staining as a marker for cellular proliferation in HDF cells. Cells were also stained with nuclear marker DAPI and imaged with a Keyence BZx-810 microscope (scale bar = 200 µm). Colocalization of Ki67 was determined using Keyence software (*n* = 3). Statistical analysis was performed by one-way ANOVA and significant *p*-values (<0.05) are indicated on the graph. (**c**) HDF cells were grown in a 35 mm plate with Ibidi μ-inserts to generate consistent gaps. Inserts were removed and HDF cells were treated with 5 ng/mL LPS for 6 h followed by treatment with Exo or Exo-G5 for 18 h. Gaps were imaged at time 0 and reimaged after 18 h and 24 h. Wound gap was measured using Image J and area was calculated in µm^2^ (*n* = 3). Statistical analysis was performed by one-way ANOVA and significant *p*-values (<0.05) are indicated on the graph. (**d**) RNA was isolated from HDF cells and SYBR Green real-time qPCR was performed using primers for IL1β and IL6; relative quantification was calculated, normalizing to GAPDH (*n* = 3). Statistical analysis was performed by one-way ANOVA and significant *p*-values (<0.05) are indicated on the graph.

We first determined cell viability using the AOPI assay in the above experimental setup. Results (Figure 3a) show that LPS decreased cellular viability. Treatment with exosomes improved cell viability, while GAS5-depleted exosomes attenuated this effect.

Next, cellular proliferation was evaluated using immunocytochemistry staining for Ki67 in the experimental setup described above. Results (Figure 3b) showed a significant decline in proliferation with LPS treatment, which was rescued by treatment with exosomes in a GAS5-dependent manner. 

We evaluated wound closure using the scratch assay in an LPS-induced inflammation environment in the experimental setup described above. A scratch assay was performed as described in Materials and Methods. Results (Figure 3c) show that LPS-induced inflammation hinders wound healing measured as closure of gap. Treatment with hASC exosomes (Exo) accelerated closure of gap, while depletion of GAS5 (Exo-G5) significantly attenuated the effect of hASC exosome-mediated repair in an inflammatory environment.

Finally, in the experimental setup described above, total RNA was extracted and SYBR Green real-time qPCR was performed to evaluate the levels of IL1β and IL6, which are markers of inflammation. Results (Figure 3d) showed that LPS increased expression of IL1β and IL6, treatment with hASC exosomes decreased its levels, while exosomes depleted of GAS5 were unable to decrease LPS-induced increase in IL1β and IL6 levels.

### 3.4. GAS5 Contained in hASC Exosomes Modulates Inflammation in a Chronic Wound Model

**Figure 4:**
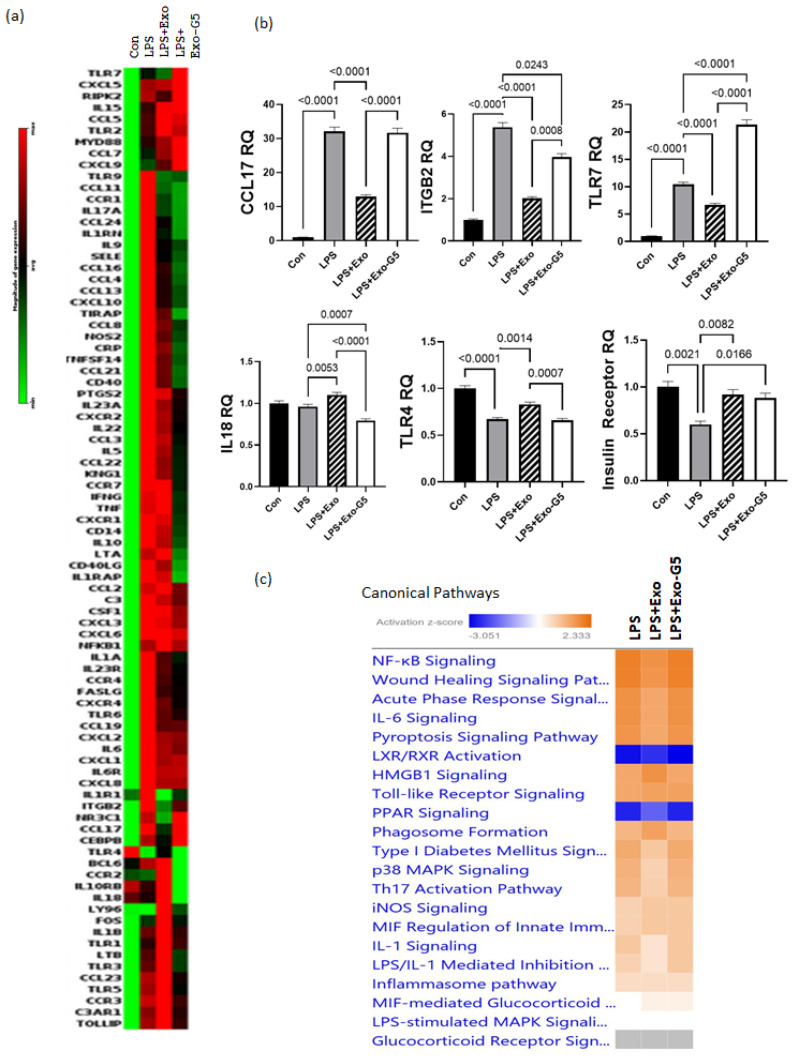

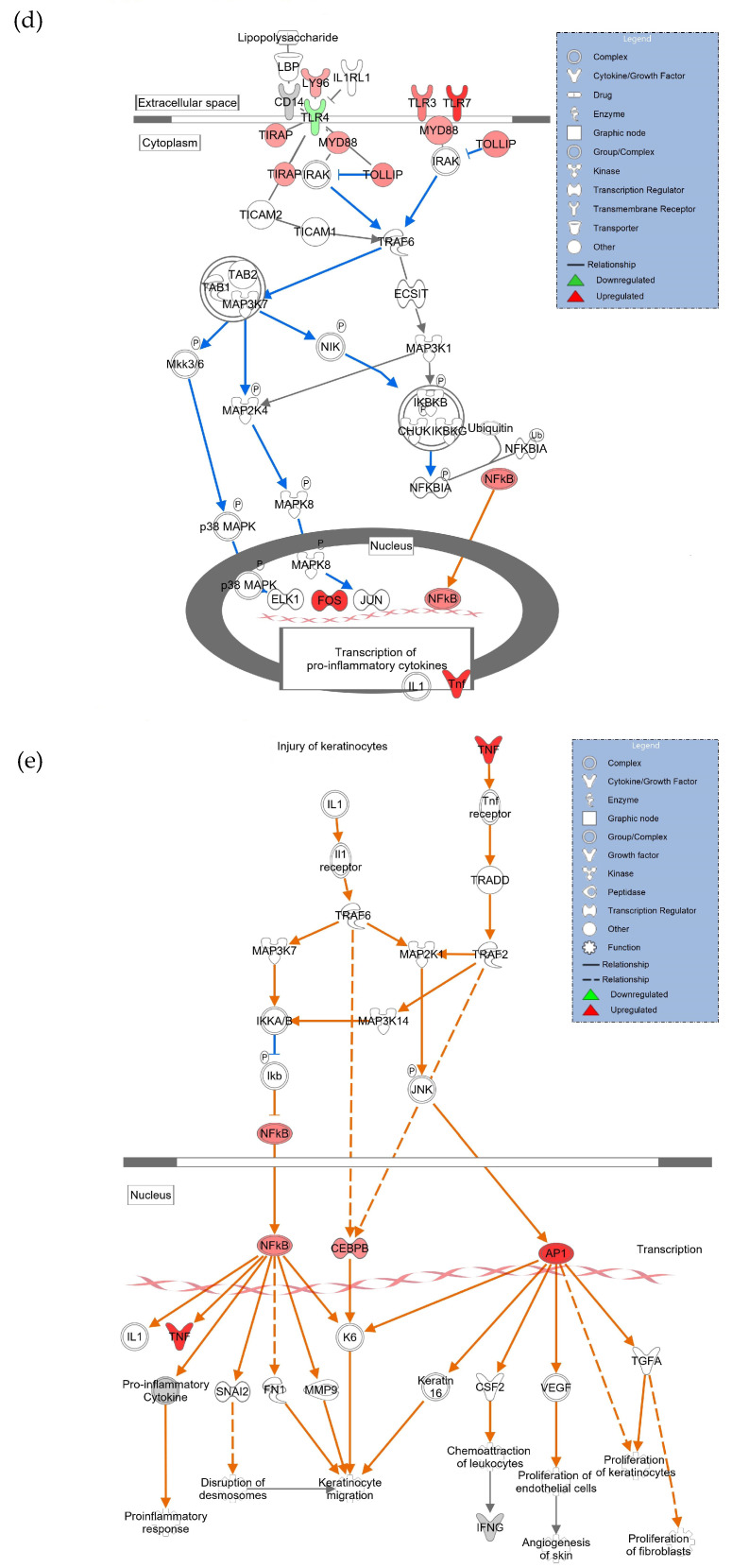
HDF cells were treated with 5 ng/mL LPS for 6 h followed by hASC exosomes or exosomes depleted of GAS5 for 18 h. LPS was maintained in the media and cells were harvested after 4 days. RNA was isolated and PCR was run using the Human Inflammation Array (Qiagen, cat #PAHS-077Z). (**a**) Using GeneGlobe online data analysis software (Qiagen), a heatmap was generated from array data showing differentially expressed genes. (**b**) Genes identified from the heatmap showing patterns correlating to LPS, Exo, and Exo-G5 treatments were verified further by real-time qPCR. Relative quantification (RQ) was determined using a control sample as reference (*n* = 3). Statistical analysis was performed by one-way ANOVA and significant *p*-values (<0.05) are indicated on the graph. (**c**) ingenuity pathway analysis was performed on array data identifying canonical pathways influenced by GAS5 presence in exosomes. Changes in (**d**) the TLR pathway and (**e**) wound healing pathway were significantly altered in response to exosome and GAS5-depleted exosome treatment.

At times, the healing of wounds is delayed or slowed, resulting in chronic recalcitrant wounds. To mimic this chronic inflammation scenario in vitro, HDF cells were pre-treated with low dose (5 ng/mL) LPS for 6 h to induce inflammation. After initiation of inflammation, cells were then treated with either Exo or Exo-G5. LPS (5 ng/mL) was maintained in the medium to mimic chronic low-grade inflammation, along with the exosome treatment for these cells for 4 days. Cells were harvested after 4 days, and RNA was isolated. To identify the genes involved in inflammation pathways that were affected with the chronic LPS treatment, we used the human inflammatory response array (Qiagen PAHS-077ZA). Figure 4a shows the differentially expressed genes compared to control samples. The results showed that most genes in the inflammatory pathway, such as those of the Toll-like receptor (TLR) family and chemokine (C-C motif and C-X-C motif) ligands and receptors and interleukins (IL), were upregulated at the end of 4 days of LPS treatment; however, some genes were downregulated at this timepoint. Significantly, TLR4 was downregulated by 33% after 4 days of low-concentration LPS treatment.

Next, we analyzed for genes that were affected by exosomes in a GAS5-dependent manner in the chronic LPS-induced inflammation environment. Towards this, we analyzed the pattern for genes whose expression compared to control changed with LPS; treatment with exosomes (Exo) reversed the LPS-induced changes, and further depletion of GAS5 in exosomes (Exo-G5) attenuated the changes of Exo in LPS samples. When pattern 1 was compared to control, the genes showed upregulation with LPS, downregulation with Exo, and upregulation with Exo-G5. The top genes with statistical significance that follow pattern 1 are TLR7, CCL17, and ITGB2. When pattern 2 was compared to control, the genes showed downregulation with LPS, upregulation with Exo, and downregulation with Exo-G5. The top gene with statistical significance that follows pattern 2 is TLR4. The genes identified in patterns 1 and 2 were then confirmed using real-time qPCR (Figure 4b). Since our previous study demonstrated that GAS5 regulated expression of the insulin receptor (IR), we also evaluated IR levels, and qPCR results showed that IR expression followed pattern 2. 

The array data were further analyzed using ingenuity pathway analysis (IPA), and Figure 4c shows that the top canonical pathways, including NF-kB signaling, wound healing signaling, acute phase response, and IL6 signaling, were changed in a GAS5-dependent manner. Since our results showed that TLR4 and TLR7 levels were changed in a GAS5-dependent manner, analysis was then performed to integrate the genes and their pathways. Figure 4d shows the intracellular location of the TLR and NF-kB pathway and the genes that were significantly affected while comparing Con vs. LPS vs. LPS + Exo vs. LPS + Exo-G5, including TLR7, TLR4, NF-kB, interleukins, and cytokines. To understand how changes in expression levels of these genes affected wound healing, we performed network analysis and the results showed how NF-kB, cytokines, and interferons integrate to promote wound healing in the skin. Overall, the data analysis showed the integration of the response to chronic LPS treatment wherein LPS binds to the TLR4 receptor along with changes in expression of TLRs which recruit cofactors and activate transcription factors and ultimately result in the expression of interferons and cytokines.

### 3.5. Depletion of GAS5 in HDF Cells Increases Expression of Toll-like Receptor 7

**Figure 5:**
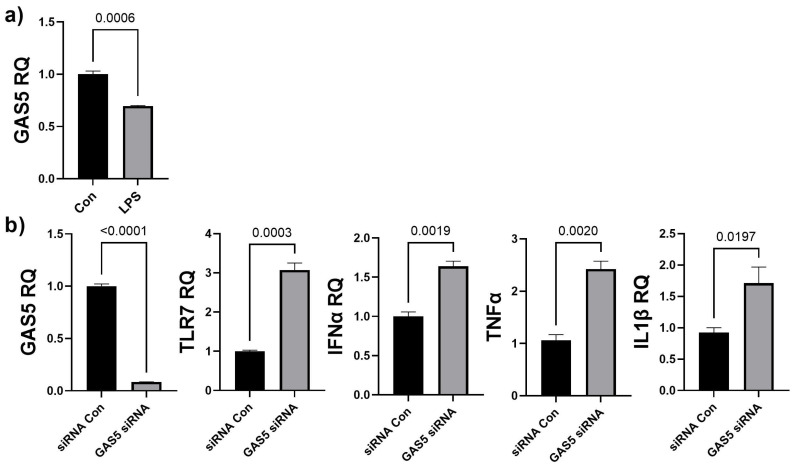
(**a**) HDF cells were treated with 5 ng/mL LPS for 4 days. SYBR Green real-time qPCR was performed using GAS5 primers. Relative quantification (RQ) was determined using a control sample as reference (*n* = 3). Statistical analysis was performed by *t*-test and significant *p*-values (<0.05) are indicated on the graph. (**b**) GAS5 was depleted in HDF cells by transfecting 25 nM GAS5 siRNA or negative control siRNA (siRNA Con) for 48 h. RNA was isolated and real-time qPCR was performed using primers specific for GAS5, TLR7, IFNα, IL1β, or TNFα. Relative quantification (RQ) was determined using a control sample as reference (*n* = 3). Statistical analysis was performed by *t*-test and significant *p*-values (<0.05) are indicated on the graph.

The results from that array showed that CCL17, ITGB2, and TLR7 were significantly increased in chronic LPS-induced inflammation. We evaluated whether chronic LPS treatment in HDF affected GAS5 levels. HDF cells were treated with LPS (5 ng/mL) for 4 days and real-time qPCR was performed using primers for GAS5. Results (Figure 5a) showed that LPS-induced chronic inflammation resulted in a 65% decrease in GAS5 levels in HDF. Hence, we evaluated whether siRNA-mediated depletion of GAS5 in HDF directly affected expression of either CCL17, ITGB2, or TLR7. GAS5 was depleted by transfecting 25 nM GAS5 siRNA (see Section 2), and real-time qPCR results (Figure 5b) demonstrate that GAS5 depletion significantly increased TLR7. CCL17 and ITGB2 levels did not change in a significant manner (not shown). Further, the qPCR results demonstrated that the levels of IFNα, IL1β, and TNFα increased with depletion of GAS5 in HDF.

### 3.6. GAS5 Contained in hASC Exosomes Mediates Repair Post-Injury in a Chronic Wound Model 

**Figure 6:**
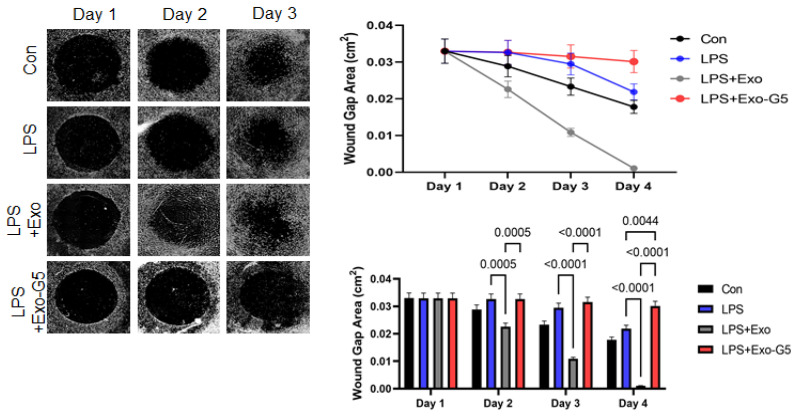
HDF cells were grown on a collagen scaffold to mimic 3D wound healing. Chronic inflammation was induced for 4 days using 5 ng/mL LPS for 6 h followed by treatment with hASC exosomes (Exo) or exosomes depleted of GAS5 (Exo-G5) for 18 h. LPS was maintained in the media along with the treatment. The 3D wound model was maintained at 37 °C and wound closure was imaged every 24 h using a Keyence BX810 microscope (*n* = 3). Wound closure was calculated each day using a Keyence’s Cell migration assay with Hybrid cell count software to calculate wound gap area. Statistical analysis was performed by one-way ANOVA and significant *p*-values (<0.05) are indicated on the graph.

To mimic the microenvironment of skin, we used a 3D wound healing model (described in Materials and Methods). After initial pre-treatment with LPS (6 h), cells were treated with Exo or Exo-G5. LPS was maintained in the medium along with the Exo or Exo-G5 treatment for these cells for 4 days to mimic accompanying chronic low-grade inflammation. Results (Figure 6) showed that chronic low-grade inflammation induced by LPS hinders the closure of wounds. Further, Exo treatment significantly accelerates wound closure compared to control in a chronic inflammatory environment. Exo-G5 treatment significantly attenuated wound closure.

## 4. Discussion

Exosomes are secreted by human adipose stem cells (hASCs) and are important mediators of the repair and regeneration post-injury attributed to hASCs [7,10,11,12,13,14]. Exosomes from hASCs are thus referred to as a stem cell-based, cell-free therapy for wound healing. The cargos of hASC exosomes contain a number of proteins necessary for targeting cells and mediating uptake and recycling, while the RNA cargos modulate gene expression in target cells. We have previously demonstrated that the noncoding RNA content of Exo drives the repair and regeneration of the Exo [7]. Our previous studies in HDF and HT22 cells and in vivo models of traumatic brain injury have demonstrated the role of MALAT1, contained in hASC exosomes, in neuronal wound healing and showed that it accounted for a portion of the regenerative properties of hASC exosomes [5,6,12].

Other studies have identified miRNAs that modulate wound healing [15,16,17]. Our results presented here demonstrated that lncRNA GAS5 was highly enriched in hASC exosomes, but its function is not yet determined. Hence, in this project we focused on elucidating the role of GAS5 contained in hASC exosomes in dermal wound healing. We evaluated the role of lncRNA GAS5 in promoting wound healing and further elucidated the pathways affected in wound healing with an underlying chronic inflammation in human dermal fibroblasts. 

To control for batch-to-batch variances, we strictly adhered to our standardization procedure. Multiple vials of hASCs from a pooled donor lot were established at the same time and served as our master cell bank and the entire project was performed using these hASCs. We carefully controlled for the stem cell markers and contents, as described in Figure 1. Such rigid adherence is necessary to translate the therapy into clinic using GMP facilities. Wounds are healed by a highly regulated process which involves the initial response of inflammation followed by proliferation and remodeling. Exosomes from hASCs (Exo) function to deliver their cargos to recipient cells and thereby affect the genomic landscape to promote healing. Hence, we undertook cellular, genetic, and physiological wound healing in vitro assays to understand the mechanisms involved in the healing of wounds with Exo treatment. 

Using the scratch assay as a model for in vitro wounds, we demonstrated that exosomes depleted of GAS5 (Exo-G5) were significantly hindered in their ability to close the wound gap compared to the Exo treatment. Other studies have identified endogenous GAS5 as pivotal in wound healing in diabetic wounds [18]. We have demonstrated that GAS5 expression is significantly lower in type 2 diabetes and further demonstrated that GAS5 regulates the expression of insulin receptor [6,19]. Other studies have shown that application of insulin to the wound also promoted healing [20,21,22]. Kino et al. showed that GAS5 regulated glucocorticoid receptor (GR) target genes by sequestering GR [23]. These studies shed light on the multifaceted role of GAS5 in regulating several signaling cascades depending on the particular cell type and environment.

In physical injury and infection, there is an immediate increase in inflammation which is critical for response and the initiation of healing. As healing progresses, inflammation is resolved and pathways mediating repair and proliferation are initiated [24,25]. Diseases such as diabetes, cardiovascular diseases, and obesity are accompanied by chronic low-level inflammation, which is a substantial risk factor for impaired wound healing and often leads to chronic wound-related sequelae. Delayed or slow wound healing is a significant problem in the clinic. Amongst the risk factors promoting recalcitrant wound healing is underlying infection in which wounds exhibit chronic low levels of inflammation. In this study, the HDF were treated with a low dose of LPS to mimic the chronic low inflammation that accompanies certain diseases [26]. LPS, usually on Gram-negative bacteria, is commonly associated with wound infections [27,28]. The innate immune system is activated as a defense mechanism against microbial infections. Members of the human Toll-like receptor family (TLR) [29] are located either on the cell surface or in intracellular vesicles called endosomes. The TLRs are expressed in several immune cells and are also expressed in other cell types, such as fibroblasts, keratinocytes, and endothelial cells. The TLRs, upon activation, dimerize and their intracellular domains recruit proteins of the MyD88 family. The cell surface receptors TLR1, TLR2, TLR4, TLR5, and TLR6 recognize the microbial pathogen-associated molecular pattern through their extracellular domain. LPS mediates the inflammatory response by activating the cell surface Toll-like receptor 4 (TLR4) which then recruits adaptor proteins MyD88, TIRAP, TRIF, and TRAM through its intracellular domain. This activates the intracellular signaling cascade which ultimately results in the expression of inflammatory cytokines. The endosomal receptors TLR3, TLR7, TLR8, and TLR9 recognize the danger-associated molecular pattern and are activated by nucleic acids, such as double-stranded or single-stranded RNA and DNA which may be released from dead cells in damaged tissues. TLR7, TLR8, and TLR9 recruit MyD88 to activate transcription factor IRF3/7 to promote the production of type I interferons. In this study, we evaluated the inflammation pathway genes comprising the TLR family and chemokine (C-C motif and C-X-C motif) ligands and receptors, and interleukins that were affected by Exo in a chronic low-grade inflammation environment. The results indicated that TLR7 expression was regulated in a GAS5-dependent manner. This indicates that release of the noncoding RNA content of hASC exosomes, and particularly lncRNA GAS5, regulated the expression of TLR7. TLR7 expressed on endosomes mediates the expression of type 1 interferons. Our results demonstrated that depletion of endogenous GAS5 significantly increased TLR7 expression and the downstream interferon and interleukin targets of TLR7. The molecular mechanism by which GAS5 can promote the expression genes is either via binding to the promoter, as shown for insulin receptor [6], or sequestering proteins, such as glucocorticoid receptors, that promote expression of target genes [23], or via sequestration of miR, which represses expression of target genes [30,31]. The exact mechanism underlying regulation of expression of TLR7 by GAS5 is being evaluated in our lab. Our unpublished scratch assay results also showed that depleting endogenous GAS5 in HDF hindered the closure of wound gaps. In this study, we focused on elucidating the pivotal role of GAS5 carried as a cargo within the exosomes and demonstrated that GAS5 was critical in promoting wound healing in a chronic inflammation environment. 

It is estimated that the cost of all wound types in the US is about $31.7 billion [32]. Our research demonstrates that exosomes derived from human adipose stem cells mediate the recovery and regeneration of wounds. The exosomes are applied topically to the wound and no transfections or carriers are required. Importantly, our results demonstrate that hASC exosomes are a viable therapeutic that accelerates healing of chronic recalcitrant wounds.

## 5. Conclusions

In summary, our results demonstrate that exosomes harvested from human adipose stem cells accelerate the healing of chronic recalcitrant dermal wounds. The lncRNA GAS5 contained in the exosomes is crucial and necessary for the therapeutic potential of hASC exosomes in treating wounds, and, further, GAS5 drives the healing of wounds in an inflammation microenvironment often seen in chronic diseases, such as diabetes and obesity. The results presented here demonstrate that hASC exosomes are a viable therapeutic that accelerates healing of chronic recalcitrant wounds.

## Data Availability

Not applicable.
